# Successful resection of a rectal gastrointestinal stromal tumor using a transperineal approach: a case report

**DOI:** 10.1186/s40792-024-02007-4

**Published:** 2024-09-10

**Authors:** Yoki Endo, Tatsunari Fukuoka, Shintaro Ozawa, Takemi Ishidate, Ken Yonemitsu, Yuki Seki, Hiroaki Kasashima, Yuichiro Miki, Mami Yoshii, Tatsuro Tamura, Masatsune Shibutani, Takahiro Toyokawa, Shigeru Lee, Kiyoshi Maeda

**Affiliations:** Department of Gastroenterological Surgery, Osaka Metropolitan University Graduate School of Medicine, 1-4-3, Asahimachi, Abeno-Ku, Osaka City, Osaka 545-8586 Japan

**Keywords:** Rectal gastrointestinal stromal tumor (GIST), Transperineal approach, Minimally invasive therapy

## Abstract

**Background:**

Rectal gastrointestinal stromal tumors (GISTs) complicate surgical approaches because of their anatomical position. We herein report a patient with rectal GIST on the anterior wall of the lower rectum, hat was successfully resected using a transperineal approach.

**Case presentation:**

This report describes a unique case of a 73-year-old man who was diagnosed with rectal GIST on the anterior wall of the lower rectum. The tumor was located within 3 cm of the anal verge, a location that would require highly invasive surgery. A transperineal approach was planned to preserve the anal function. Under general anesthesia, the patient was placed in a lithotomy position and a Mercedes-Benz incision was made in the perineum. Excision of the tumor was performed. The post-operative course was uneventful, and the patient remained free from recurrence.

**Conclusion:**

This case highlights the importance of performing minimally invasive and safe surgery. With some surgical refinements, a transperineal approach may be an option for surgical procedures in patients with rectal GIST on the anterior wall of the lower rectum.

## Background

Rectal gastrointestinal stromal tumors (GISTs) are the next most common after those of the stomach, small intestine, and colon, with a reported incidence of approximately 3–5% [1]. In principle, the treatment of rectal GIST is surgical, but there are many cases in which anorectal preservation is not possible due to difficulty in securing the field of view or in manipulating the deep pelvic region [2].

We herein report a case of rectal GIST that was successfully resected using a transperineal approach and review the literature.

### Case presentation

**Fig. 1:**
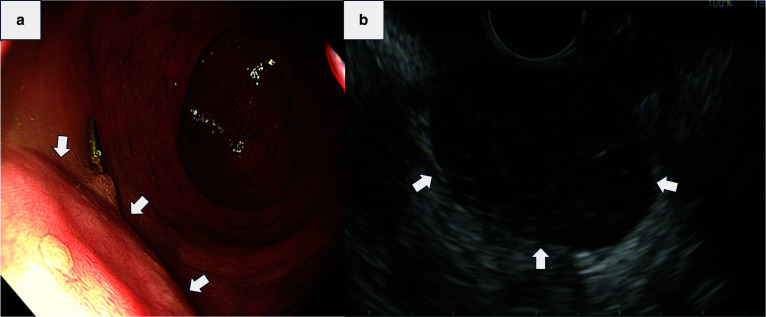
Colonoscopy and transrectal ultrasonography. **a** Colonoscopy showed a bulge that probably originated in the submucosal layer. **b** Transrectal ultrasonography showed a 28-mm submucosal tumor with clear borders and ring contouring

**Fig. 2:**
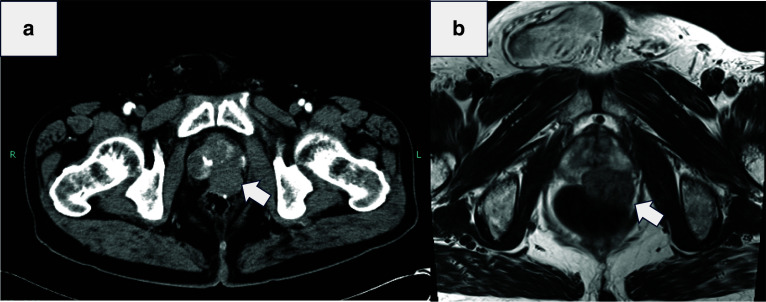
CT and MRI findings. **a** CT showed a homogeneously contrast mass on the anterior rectal wall. No distant metastasis or enlarged lymph nodes were observed. **b** T2 weighted MRI demonstrated that the tumor was compressing the prostate, but there was a clear border between the prostate and the tumor

A 73-year-old man was referred to our hospital for a further examination and treatment after a rectal mass was incidentally detected on magnetic resonance imaging (MRI). The patient had neither constitutional symptoms nor any history of note. A digital rectal examination revealed a small mass on the anterior wall of the rectum 3 cm from the anal verge. The mass was elastic, hard, and non-mobile, with a smooth surface. Routine blood tests, serum chemical analyses and tumor marker analyses revealed no abnormalities. Colonoscopy and transrectal ultrasonography revealed normal mucosal elevation in the anterior rectal wall (Fig. 1a, b). The mass was 28 mm in diameter and contiguous with the fourth layer, with clear borders and a well-defined contour. A histological examination of the rectal biopsy sample via the rectum led to a diagnosis thanks to immunohistochemical positivity for C-kit, CD34, and DOG1. Abdominal contrast-enhanced computed tomography (CT) showed a mass with a uniform contrast effect without internal calcification or a hemorrhagic component on the anterior wall of the rectum (Fig. 2a). No distant or lymph node metastasis was observed. T2 weighted MRI demonstrated a mass on the anterior wall of the rectum without any invasion of the prostate (Fig. 2b). The tumor was relatively small and had clear margin without invasion to the surrounding tissues. Then we decide to remove the tumor using a transperineal approach. As part of his bowel preparation, the patient took a colon stimulant laxative two days before surgery and kanamycin and metronidazole one day before surgery.

**Fig. 3:**
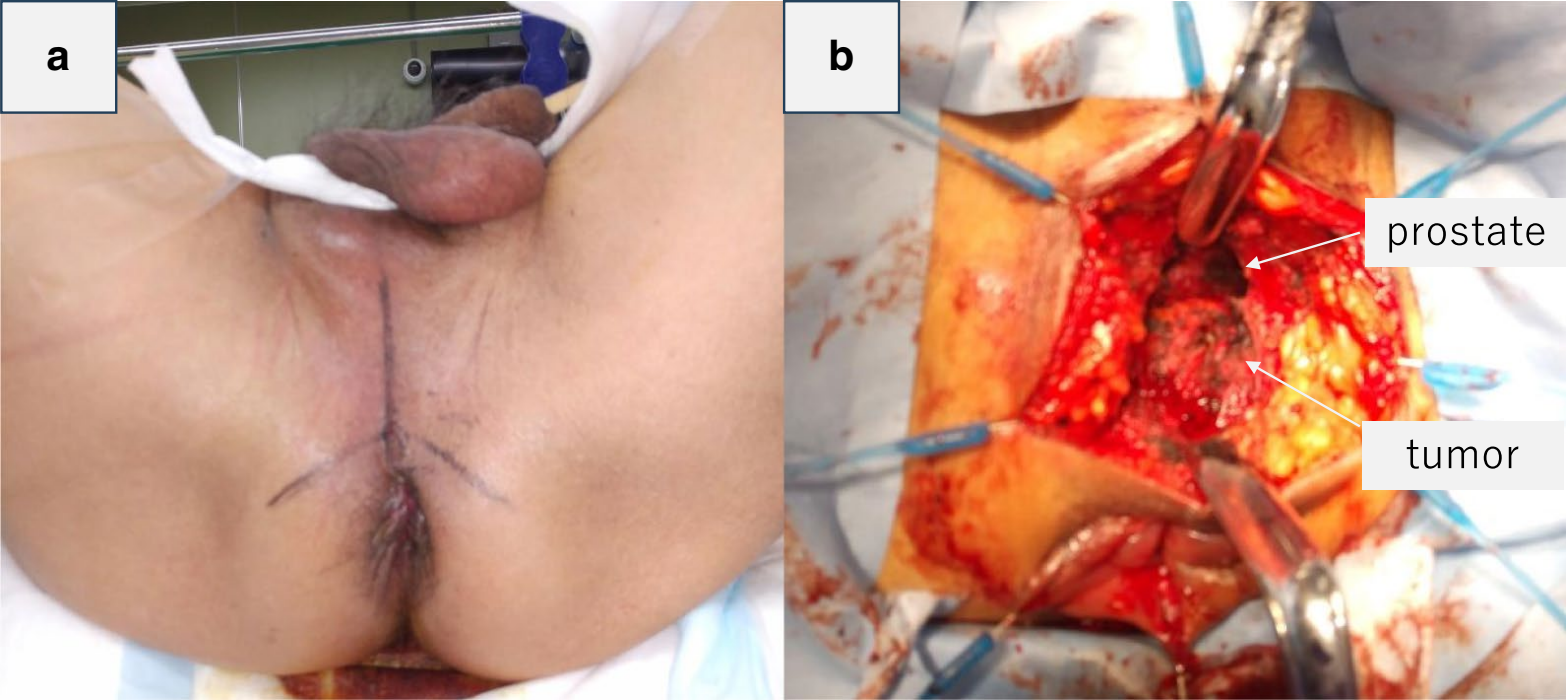
Intraoperative findings. **a** Mercedes-Benz incision was made on the perineum. **b** The boundary between the prostate and the tumor was confirmed to have a good field of view

**Fig. 4:**
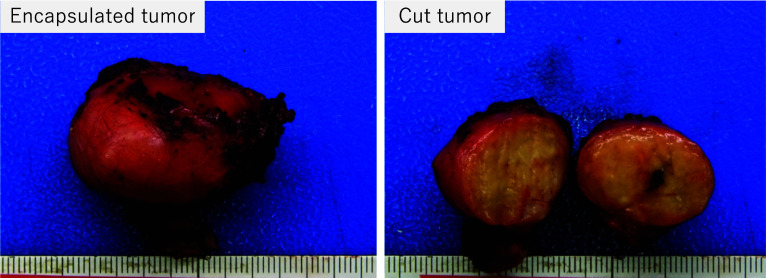
Gross pathological examination findings. A gross pathological examination of the specimen showed a 34 × 29 × 24-mm elastic-hard mass

Under general anesthesia, the patient was placed in the lithotomy position. A Mercedes-Benz incision was made in the perineum (Fig. 3a). The transverse perineal muscle was identified by careful dissection to avoid damaging the prostate and urethra. The border with the prostate was dissected to expose the cephalic edge of the tumor (Fig. 3b). The rectourethral muscle was resected on the rectal side to avoid damaging peripheral branches of the pelvic plexus such as the cavernous nerve. Full-thickness resection was performed along with marking of the tumor while confirming the tumor under the speculum from the transanal view. The rectal wall was closed horizontally using primary suturing. Suturing was added along with the rectourethral muscle and supporting tissue around the rectum. Two drains were inserted into the outer layer of the closed rectal wall and the rectum transanally. A diverting stoma was not performed. The surgery time was 158 min, with minimal bleeding. The resected tumor was a 34 × 29 × 24-mm elastic-hard mass (Fig. 4). A histopathological examination revealed spindle-shaped cells that proliferate in bundles with positive staining for C-kit and CD34, and Ki-67 index of 0.3%. A diagnosis of GIST with low-risk behavior was made.

The post-operative course was uneventful. On postoperative day 6, the patient resumed eating and the transanal drain was removed. On postoperative day 7, the subcutaneous drain was removed. On postoperative day 16, he was discharged. Neither local recurrence nor distant metastasis was noted during the two-year follow-up period.

## Discussion

GISTs are the most common stromal tumors of the gastrointestinal tract, with a reported incidence of 7–19 per million per year [3]. GISTs can occur anywhere in the gastrointestinal tract, and rectal GISTs are the next most common after those of stomach, small intestine, and colon, with a reported incidence of approximately 3–5% [1]. Surgery is the first choice of treatment for complete resection [4]. Systematic lymph node dissection is not recommended because it does not contribute to the prognosis. Complete resection with negative histological margins is of paramount importance [5]. Neoadjuvant chemotherapy with imatinib is recommended for cases in which R0 resection is difficult, and many articles have discussed its effectiveness. However, in the present case, the largest diameter was only 33 mm, and the compression of the surrounding organs was mild, so we diagnosed complete resection was possible and did not perform chemotherapy.

Complete tumor removal, while preserving the capsule, is the goal of surgical excision. Rectal GIST often occurs below the peritoneal reflection, and abdominoperineal resection or low anterior resection is often the surgical choice. Recently, intersphincteric resection (ISR) has been reported to preserve the anal function [6]. However, all of these procedures are highly invasive and prone to anorectal dysfunction. Therefore, local excision should be considered whenever possible. There are some reports in the literature describing transcoccygeal, transanal, transvaginal, and transperineal approaches for local excision of GIST located in the lower rectum. The transcoccygeal approach is effective for lesions located on the posterior wall more than 5 cm from the anal verge [7]. However, the risk of complications is higher than with other approaches, especially with postoperative fistula formation reported in 21% of patients [8]. The transanal approach is considered the most minimally invasive approach, but is suitable for lesions up to 3 cm from the anal verge [9]. However, this approach is difficult to select because it is difficult to secure the field of view and is not a common surgical approach for surgeons. Another problem is the difficulty in securing the resection margin to the prostate or oral side, which may be more difficult in cases where the tumor protrudes outside the lumen. The transvaginal approach is considered less likely to have complications than the transanal approach, and there have been reports discussing its efficacy [10, 11]. The transperineal approach is relatively minimally invasive and allows for preservation of the anal function but carries a risk of prostate and urethral injury and the possibility of gonadal dysfunction. The surgical strategy depends largely on the location and size of the tumor and should be determined carefully with various imaging studies, such as MRI. In the present case, the tumor was located approximately 3 cm from the anal verge on the anterior wall of the rectum, protruded outside the lumen, and was in contact with the prostate. Therefore, we used a transperineal approach.

To our knowledge, there have been only seven reported cases of transperineal resection of rectal GIST [12–18] (Table 1). The median age was 58.9 years old, with 6 men and 1 woman. The mean tumor diameter was 38 mm, and the mean distance from the anus was 29 mm. In all reports, the patient had a good course, without major complications or recurrence.

**Table 1:** Analysis of reported cases of rectal GIST through a transperineal approach

No	Author	Age (years)	Sex	Tumor diameter (mm)	Dist (mm)	Incision	Days from surgery to discharge (days)	Complication
1	Hamada [12]	60	M	32	32	Transverse	Not written	Not written
2	Marumori [13]	44	M	45	45	Hemispherical	Not written	Not written
3	Babaya [14]	60	M	24	24	Hemispherical	43	SSI, UTI
4	Kinoshita [15]	61	M	20	20	Hemispherical	14	none
5	Yasuda [16]	77	F	42	42	Transverse	8	none
6	Mizutani [17]	57	M	63	63	Spherical (tracing the tumor)	13	none
7	Inna [18]	53	M	Not written	Not written	Transverse	Not written	none
8	Our case	73	M	28	3	Mercedes-Benz	16	none

M, male; F, female; Dist, distance between the anal verge and tumor; SSI, surgical site infection; UTI, urinary tract infection

Three incisions of the perineum were transverse, three hemispherical, and one spherical, whereas the Mercedes-Benz incision was made only by our group. As mentioned above, the transperineal approach has a limited field of view and there is a possibility of injury to adjacent organs. By adding a longitudinal incision, as in the Mercedes-Benz incision, the prostate and urethra can be located under a good field of view. This technique allowed the surgery to be performed more safely than other incision methods. In terms of surgical technique, it is essential to minimize injury to the levator ani muscles and proceed with dissection while carefully checking the boundaries with the urethra and prostate. With this technique, even if the prostate gland is involved, it is possible to perform a combined resection of the prostate gland with the same field of view.

In the present case, the patient underwent partial transperineal resection and was discharged on the postoperative day 16 without major complications. The patient was free from recurrence two years after surgery.

## Conclusion

We encountered a case of a rectal GIST that was successfully resected using a transperineal approach. With some surgical refinements, this transperineal approach can be safely performed for GISTs on the anterior wall of the rectum.

## Data Availability

Not applicable.

## Abbreviations

GIST: Gastrointestinal stromal tumor
CT: Computed tomography
MRI: Magnetic resonance imaging
ISR: Intersphincteric resection
